# Eicosapentaenoic Acid Extraction from *Nannochloropsis gaditana* Using Carbon Dioxide at Supercritical Conditions

**DOI:** 10.3390/md17020132

**Published:** 2019-02-22

**Authors:** Antonio Molino, Maria Martino, Vincenzo Larocca, Giuseppe Di Sanzo, Anna Spagnoletta, Tiziana Marino, Despina Karatza, Angela Iovine, Sanjeet Mehariya, Dino Musmarra

**Affiliations:** 1 ENEA, Italian National Agency for New Technologies, Energy and Sustainable Economic Development, Department of Sustainability-CR Portici, P. Enrico Fermi, 1, 80055 Portici, Italy; 2 ENEA, Italian National Agency for New Technologies, Energy and Sustainable Economic Development, Department of Sustainability-CR Trisaia, SS Jonica 106, km 419+500, 7026 Rotondella, Italy; maria.martino@enea.it (M.M.); vincenzo.larocca@enea.it (V.L.); giuseppe.disanzo@enea.it (G.D.S.); anna.spagnoletta@enea.it (A.S.); 3 Institute on Membrane Technology, National Research Council (ITM-CNR) Via Pietro Bucci, Cubo 17C, 870 36 Rende, Italy; tiziana.marino@yahoo.it; 4 Department of Engineering, University of Campania “L.Vanvitelli”, Real Casa dell’Annunziata, Via Roma 29, 81031 Aversa, Italy; karatza@irc.cnr.it (D.K.); angela.iovine@unicampania.it (A.I.); sanjeet.mehariya@unicampania.it (S.M.); dino.musmarra@unicampania.it (D.M.)

**Keywords:** microalgae, *Nannochloropsis gaditana*, lipids, eicosapentaenoic acid (EPA), Supercritical-CO_2_ fluid extraction, pharmaceutical, nutraceutical

## Abstract

This research shows that carbon dioxide supercritical fluid (CO_2_-SF) is an emerging technology for the extraction of high interest compounds for applications in the manufacturing of pharmaceuticals, nutraceuticals, and cosmetics from microalgae. The purpose of this study is to recover fatty acids (FAs) and, more precisely, eicosapentaenoic acid (EPA) from *Nannochloropsis gaditana* biomass by CO_2_-SF extraction. In the paper, the effect of mechanical pre-treatment was evaluated with the aim of increasing FAs recovery. Extraction was performed at a pressure range of 250–550 bars and a CO_2_ flow rate of 7.24 and 14.48 g/min, while temperature was fixed at 50 or 65 °C. The effect of these parameters on the extraction yield was assessed at each extraction cycle, 20 min each, for a total extraction time of 100 min. Furthermore, the effect of biomass loading on EPA recovery was evaluated. The highest EPA extraction yield, i.e., 11.50 mg/g, corresponding to 27.4% EPA recovery, was obtained at 65 °C and 250 bars with a CO_2_ flow rate of 7.24 g/min and 1.0 g biomass loading. The increased CO_2_ flow rate from 7.24 to 14.48 g/min enhanced the cumulative EPA recovery at 250 bars. The purity of EPA could be improved by biomass loading of 2.01 g, even if recovery was reduced.

## 1. Introduction

Microalgae are unicellular organisms that, in the presence of sunlight, convert carbon dioxide (CO_2_) into biomass containing high-value products, such as fatty acids (FAs) and carotenoids. These compounds find applications within the food and feed, cosmetic, and pharmaceutical industries. The cultivation of microalgae shows several advantages when compared to terrestrial crops. In fact, microalgae exhibit shorter cultivation time, higher biomass production, no requirements of fertilizers and pesticides, lesser land use, and higher CO_2_ fixation rate with respect to crops. Microalgae are able to fix around 1.8 kg of CO_2_ per kg of dry biomass that comes from different sources, including atmosphere, industrial exhaust gases, and upgrading plants for biomethane production. In this context, cultivation of microalgae can be an economical and eco-friendly approach to fulfill the demand of nutritious food and simultaneously reduce the greenhouse gas in line with the concept of circular economy [1,2,3,4]. 

Microalgae are able to produce FAs in their saturated, mono unsaturated and polyunsaturated form, depending on the kind of bonds in their chain. Among the several microalga genus, *Nannochloropsis* is able to accumulate higher lipids and FAs content [5,6,7,8]. *Nannochloropsis* is unicellular, with coccoid cells and polysaccharide cell walls, and it belongs to the photoautotrophic group of microalgae in the Eustigmatophyceae stramenopile family that are found in fresh, brackish, and sea waters. *Nannochloropsis* cells reproduce asexually, dividing to yield two daughter cells that then shed their mother cell wall [7,9,10,11,12]. In this genus, *N. gaditana* can synthesize the maximum amount of polyunsaturated fatty acids (PUFAs), which represent a significant portion of lipids. PUFAs are contained inside the cells and they are linked to glycerol molecules to constitute glycolipids that have a structural function for the cellular membrane. PUFAs are also classified as ω-3, ω-6, and ω-9, based on the position of the last double bond from the terminal carbon that is defined as ω carbon. PUFAs, specifically ω-3 and ω-6 fatty acids, are defined as essential fatty acids because the human body is not able to synthesize these substances that are very important for human health. In particular, these essential substances are α -linolenic acid (ALA, ω-3) and linoleic acid (ω-6), which are only produced by vegetables as plants and microalgae. Other important compounds for human health are eicosapentaenoic acid (EPA, ω-3), docosahexaenoic acid (DHA, ω-3), and arachidonic acid (ARA, ω-6). These compounds have several benefits on human health, as they contribute to the prevention of both cardiovascular and inflammatory diseases, to the mental health in adults, and to the development of the brain of the fetus. They are also important for children in the early years of life for their unique pharmaceutical properties [13]. The World Health Organization (WHO) suggests the correct dose of 250 mg/day of EPA and DHA for adults, while 100–200 mg/day of EPA and DHA for pregnant women and children [14,15] Therefore, a suitable source of these compounds need to be found out to supplement the food for human consumption. *N. gaditiana* species, being able to accumulate significant amount of EPA (up to 4.3% wt/wt [16]), have been suggested to be among the most promising microalgae for EPA commercial applications. 

Moreover, the complete extraction of high purity EPA from *N. gaditana* biomass is necessary to use it as a source of ω-3 in the food and pharmaceutical industry. Therefore, the biomass rigid cell walls can be disrupted to enhance the recovery of FAs and, for this purpose, mechanical pretreatments, such as bead milling, are commonly used [17,18,19]. The common FAs extraction methods are based on the use of solvents, acids, edible oils, enzymes, pressurized liquids, and carbon dioxide supercritical fluid (CO_2_-SF) [20,21,22,23,24,25,26,27,28,29,30,31]. CO_2_-SF extraction is preferable for the extraction of thermolabile compounds due to the lower adopted temperature and faster extraction, which allow for less contact time with heat and pressure [32]. In addition, CO_2_-SF has low viscosity, low surface tension, high diffusivity, and good density and it is also non-toxic, non-flammable, cheap, widely available, chemically inert under several conditions, and gaseous at normal pressure and temperature, eliminating the step of solvent evaporation after extraction [33]. Moreover, CO_2_ provides a non-oxidizing atmosphere during extraction, therefore avoiding the degradation of extracted compounds [26,34]. Although many studies explored various methods of extraction and downstream processing of PUFAs from *N. gaditana* biomass for commercial applications, only green extraction technologies and solvents should allow for the extraction of these compounds for a direct application in the food industry [35,36,37,38,39].

In this study, the *N. gaditana* marine microalgae biomass was selected for the extraction of PUFAs and, in particular, EPA by using CO_2_-SFE technology after mechanical cell disruption. The purpose of this study was to evaluate the purity and recovery of EPA for direct use in pharmaceutical, neutraceutical, and cosmetic industries. 

## 2. Materials and Methods

### 2.1. Microalgal Biomass and Chemical Composition 

Lyophilized *N. gaditana* biomass that was provided by Algalimento, Spain, with a mesh particle size of about 5–25 μm, was used to carry out experimental activity. The biomass was stored at −20 °C in a vacuumed plastic bag to avoid degradation until further utilization and brought to room conditions before use. The chemical composition of lyophilized *N. gaditana* in terms of humidity, ash, total dietary fiber (TDF), carbohydrates, proteins, FAs, saturated fatty acids (SFAs), monounsaturated fatty acids (MUFAs), PUFAs, and specifically EPA was carried before extraction and reported in Table 1, which was analyzed using standard methods that are reported in our previous publication [40]. The investigated microalgal species was characterized by a lipids content of about 164.5 mg/g of dry biomass, which corresponds to ≅16.45% *w*/*w* of dry biomass. 

### 2.2. Chemicals 

Rivoira, Italy provided the O_2_ (99.999% purity) that was used for supercritical fluid extraction. The standards (FAs) that were used for the GC calibrations were of analytical grade and purchased from Sigma Aldrich, USA. All of the solvents used were of HPLC grade and purchased from Sigma Aldrich, USA.

### 2.3. Mechanical Pre-Treatment of Biomass

In order to improve FA recovery, the mechanical pre-treatment conditions were optimized in terms of biomass and diatomaceous earth mixing ratio, rotation speed, and time using a Retsch PM200 planetary ball mill [18]. The jars of the mill were filled with 2 g of *N. gaditana* biomass and diatomaceous earth (DE), with a mixing ratio of 0.5, 1.0, and 2.0 DE/biomass. In the first experiment, pretreatment was performed for 5 min at different rpm (200, 300, 400, 500, and 600 rpm).

In the second series of experiments, the optimum ratio of DE/biomass and rpm was determined to optimize the pretreatment time from 2.5 to 25 min (2.5, 5.0, 7.5, 10, 15, 20, and 25 min). The mechanical pretreatment of biomass extraction was carried using accelerated solvent extractor (ASE) with n-hexane as solvent at 50 °C and 100 bar, which have the same polarity with lipids. Using two extraction cycles each extraction test was carried; each one being 10 min for total extraction of 20 min. 

### 2.4. CO_2_ Supercritical Extraction Experiments

CO_2_-SF extraction was achieved by using a bench scale extraction unit, as described in our previous study [23,40]. The extraction unit had a heating capacity up to 250 °C and CO_2_ compression capacity up to 680 bars. The extraction unit can control the inlet and outlet pressure with, accuracy of 0.6 mbars, and the CO_2_ flow rate was controlled using a flow meter LPN/S80 ALG 2.5, Sacofgas, Italy. The inlet flow rate was adjustable until 25 mL/min and controlled using the expanded gas. Temperature was monitored using thermocouples, where micrometric valves control the inlet and outlet flow streams. The extraction cylindrical vessel had a capacity of 50 mL (D = 1.35 cm, H = 35 cm), which was filled with pretreated biomass, including diatomaceous earth, and 44 gr glass beads of 3 mm to increase the contact of carbon dioxide with microalgae, and at the same time avoid the biomass caking.

**Figure 1:**
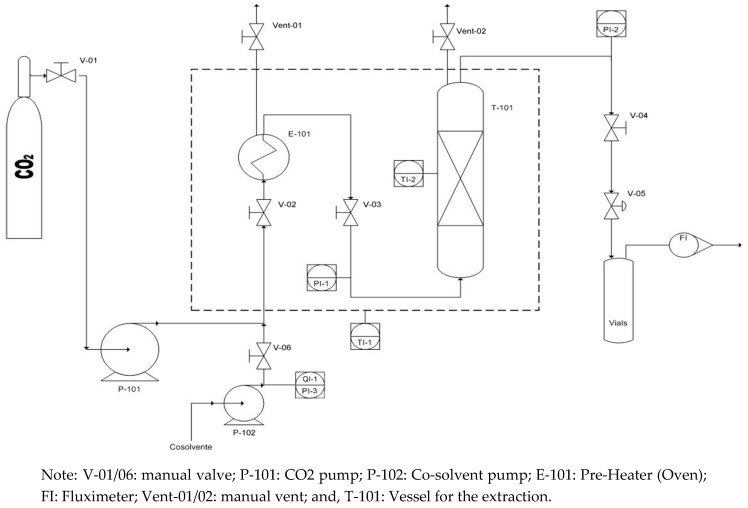
P&ID of bench scale CO2-SFE unit.

Furthermore, at the bottom of extraction vessel, metal frit filters were used with a pore diameter of 5 μm. The extraction unit was equipped with acoustic and visual high pressure alerts and, as a primary security system, a rupture disk is installed. All of the parameters are controlled with a Distributed Control System (DCS). The sketch and P&ID of bench scale extraction unit is reported in Figure 1.

The effect of the various operative conditions, such as extraction time in the range 20–100 min, pressure (P) in the range 250–550 bar, CO_2_ flow rates 7.24 and 14.48 g/min, temperature (T) 50 and 65 °C, and biomass loadings 1.01 and 2.01 g, on the extraction of FAs from *N. gaditana* was investigated. 

The influence of the adopted operating conditions was studied on FA species (SFAs, MUFAs, PUFAs, and EPA) recovery and is expressed, as follows:

(1) $$\mathrm{Recovery}(\mathrm{mg}/g)=\;W_{C,i}/W_{M}$$

where *W_C,i_* is the weight of FAs class (mg) extracted; and, *W_M_* is the weight of microalgae on dry basis (g). Moreover, for each class, the recovery was compared with respect to the theoretical content.

The recovery percentage and purity of EPA was derived from Equations (2) and (3) [23].

(2) $$\mathrm{Recovery}\left(\%\right)=\;W_{B}/W_{T}\times 100$$

(3) $$\mathrm{Purity}\;\left(\%\right)=W_{B}/W_{E}\times 100$$

where *W_B_* is the weight of EPA extracted (mg); *W_T_* is the theoretical weight EPA (mg); *W_E_* is the total weight of the extract (mg).

Each experimental condition was investigated three times, and for each condition, the standard deviation (SD) value was calculated. After the CO_2_-SF extraction, the extracts were stored in the dark at −80 °C before analyzing the total FAs and EPA contents using GC-FID.

### 2.5. Analytical Methods 

The extracts that were obtained after the CO_2_-SF extraction were transesterified according to the indications given in the standard method UNI ISO 12966-2 [41,42]. NaOH solution in methanol (0.5 M, 6 mL) and a spatula of boiling chips were added to a known quantity of extract (about 100 mg). The sample was transferred to a 50 mL one-mark volumetric Erlenmeyer flask that was connected to a reflux condenser to boil the sample for about 10 min. At the end of boiling, the apparatus was removed from the heat source and 6 mL of n-hexane was added from the top of the condenser and then 7 mL of the BF_3_ catalyst in methanol (14%) (B1252 Aldrich). The sample was boiling again for 30 min and 5 mL of isooctane were added at the end of the reaction. A 20 mL sample of a saturated NaCl solution were added and swirled, and a second aliquot of saturated NaCl solution was added until the neck of the flask. The total upper layer (2−4 mL) was taken and then transferred to a GC glass vial. The chromatographic analysis was carried out using a 7820A GC-FID that was equipped with an HP-88 100 mt × 0.25 mm × 0.2 µm column. This chromatographic column that was produced by Agilent is composed of a high polarity bis (Cyanopropyl) siloxane stationary phase and it was chosen for its high resolution of positional and geometric isomers of fatty acid methyl esters. According to the chromatographic conditions reported in the standard method UNI ISO 12966-4 [42] the temperature of the injector was maintained at 250 °C as well as detector temperature. The column was maintained at 120 °C for 5 min and was followed by temperature ramping at 4 °C/min to 240 °C and held for a further 10 min at 240 °C. Nitrogen (purity ≥ 99.9999%) was used as carrier gas with a linear velocity of 30 cm/s (flow rate approx. 1.0 mL/min) and split ratio of 1:100. The sample injection volume was of 1 µL. The FAs characterization was carried out for each extraction condition and an internal analytical standard of the tricosanoic acid (C:23) was used for the quantification of fatty acid methy esters. A mixture of 37 fatty acid ethyl esters (C4–C24) (Supelco FAME 37, CRM47885) was purchased from SIGMA-Aldrich (H5149), St Louis, MO, USA and was used for the quantitative analysis.

## 3. Results and Discussion

### 3.1. Effect of Mechanical Pre-Treatment on Fatty Acid Recovery 

**Figure 2:**
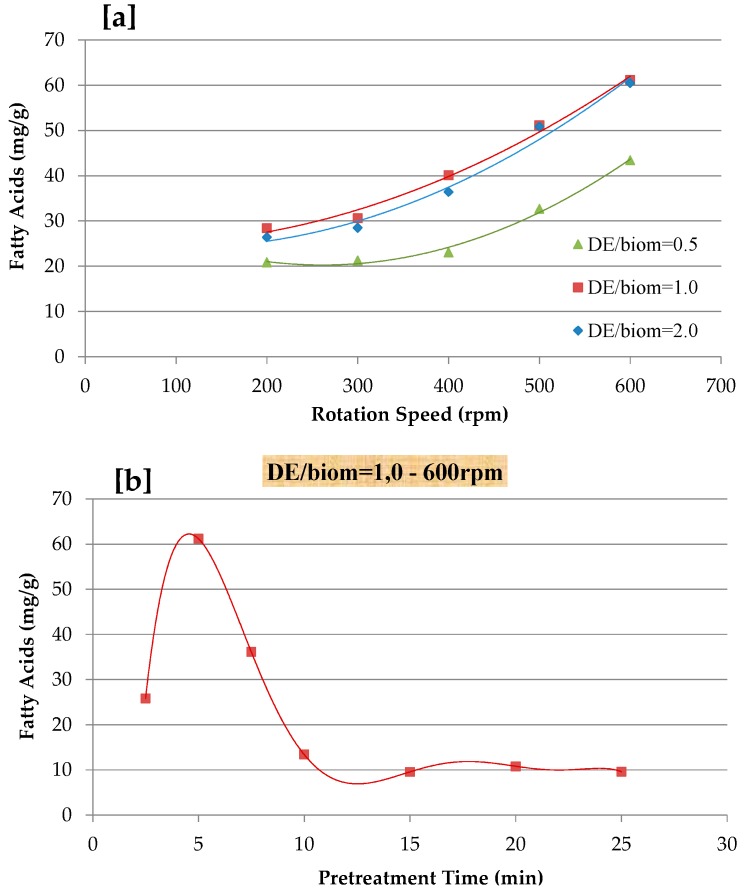
Effect of mechanical pre-treatment on fatty acids recovery; (**a**) effect of different diatomaceous earth (DE)/biomass mixing and rotation speeds; and, (**b**) effect of pre-treatment time at 600 rpm and DE/biom = 1.0.

One of the key features of microalgae is the rigidity of its cell wall, while damage to their cell wall permits further CO_2_-SF to reach the selective compound. There are several cell disruption methods, such as mechanical, physical, chemical, and enzymatic approaches [3,19,43,44,45]. Lee et al. [17] reported a study to compare different microalgal biomass pretreatment methods and suggested that mechanical disruption methods be considered as highly energy efficient approaches when conducted under laboratory conditions. Therefore, mechanical pre-treatment was considered for the extraction of FAs from *N. gaditana* by varying the grinding speed (200–600 rpm), pretreatment time (5–25 min), and mixing ratio of diatomaceous earth (DE) and *N. gaditana* biomass (0.5–2.0 DE/biomass), as reported in Figure 2. As shown, the extraction yield of FA gradually increased with an increasing of grinding speed and by varying the DE/biomass ratio. The result showed that a lower extraction yield of FA was obtained at 0.5 DE/biomass and 200 rpm for 5 min, while the mechanical pretreatment of 1.0 DE/biomass mixing ratio for 5 min at 600 rpm resulted in maximum FA recovery. In terms of pretreatment time (2.5–25 min), an optimum pretreatment of 1.0 DE/biomass mixing ratio at 600 rpm was explored (Figure 2a). The effect of pretreatment time is reported in Figure 2b and the highest extraction yield was at 5 min with a grinding speed of 600 rpm and gradually decreased until the pretreatment time of 25 min. Before the CO_2_-SF extraction processes, all of the samples were mechanically disrupted at 600 rpm at 5 min with 1.0 DE/biomass mixing ratio. Safi et al. [37] indicated that bead milling pretreatment could increase the total extraction yield from *Chlorella vulgaris* by 16%, with similar results being reported for high-pressure disruption pretreatment [36]. Elst et al. [46] reported that the freeze-drying pretreatment of *Nannochloropsis* sp. enhance the final extracted yield by two-fold. The high pressure homogenization (HPH) of *N. oculata* did not affect the lipids extraction yield, when extracted with the method of Bligh and Dyer, whereas lipids that were extracted with the soxhlet method ranged between 8.2 and 16.2% [47]. Cheng et al. [48] suggested that the mechanical pretreatment of *Pavlova* sp. using bead-beating improves the FAME yield during CO_2_-SF.

Therefore, the pretreatment of *N. gaditana* biomass was required to maximize FA recovery to minimize the overall all cost of the extraction technologies. An ideal biomass disruption process cannot only assist internal product extraction by removing cell wall barriers, moreover, it is also able to increase mass transfer and simplify downstream processing. 

### 3.2. Effect of Extraction Pressure on Lipids Recovery 

Due to the non-polar property of CO_2_ molecules, CO_2_-SF is considered to be a suitable solvent for the extraction of lipids. Moreover, it was reported that CO_2_-SF was selective for lipids, such as SUFA, MUFA, and PUFA [49,50,51,52]. It is well-known that the solubilization of CO_2_-SF can be modified by varying pressure, temperature, CO_2_ flow rate, and extraction time. The extraction yield varies strongly with the operating conditions, which could lead to maximum (i.e., 100%) lipid recovery [49,50].

In Table 2, the experimental conditions and the main results are summarized: i.e., extraction yield and total lipid extraction yield. The maximum crude extraction yield, as obtained by CO_2_-SF extraction of *N. gaditana*, was 122.96 mg/g, this value was obtained at 550 bars and 65 °C with 5 extraction cycle (each for 20 min). On the other hand, the maximum lipid extraction yield was attained at 250 bars and 65 °C with 100 min extraction time. These results showed that increasing pressure could improve the extraction yield, while the best selectivity for lipid recovery was attained at lower pressure. Andrich et al. [49] suggested obtaining good extraction efficiencies of lipids, it is then advised to work under high pressures and temperatures. Cheung [53] obtained lipid yields of 67.1 mg/g dry weight from *Hypneacharoides* at 50 °C and 380 bars with a CO_2_ flow rate of 1 mL/min during an extraction duration of 60 min. Increasing the extraction temperature results in Table 2 reflects a slight increase in the extraction yield that may be due to an increase in the vapour pressure of the solutes and the increase in the diffusivity of the CO_2_. By changing the CO_2_ flow rate from 7.24 g/min to 14.48 g/min, the effect that prevailed was the increase in the density of the solvent and this was not compensated by the decrease in the diffusivity and the vapour pressure of the solutes to be extracted [22].

**Figure 3:**
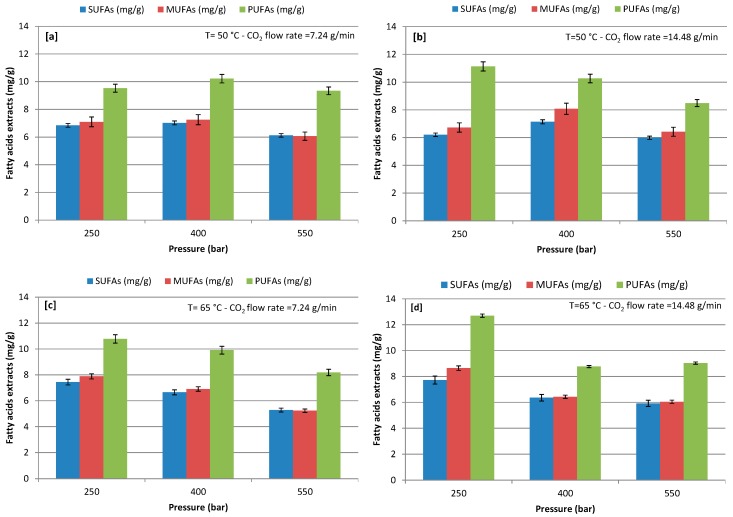
Effect of different pressure on recovery of different class of fatty acids (FAs); (**a**) at 50 °C and CO_2_ flow rate of 7.24 g/min; (**b**) at 50 °C and CO_2_ flow rate of 14.48 g/min; (**c**) at 65 °C and CO_2_ flow rate of 7.24 g/min; and, (**d**) at 65 °C and CO_2_ flow rate of 14.48 g/min.

At varying extraction conditions, concentrations of SFA, MUFA, and PUFA were detected in the FAs at an extraction pressure of 250–550 bars with a CO_2_ flow rate of 7.24 and 14.48 g/min at 50 and 65 °C (Figure 3). The quantitative analysis of the FAs highlighted the presence of major PUFA, while SUFA was the lowest and MUFA was slightly higher than SUFA during the use of CO_2_-SF extraction in all of the extractive conditions. Extraction pressure showed significant influence on the recovery of a different class of FAs; increasing pressure from 250 to 550 bars negatively influenced the extraction of FAs, except extraction at 400 bars with a CO_2_ flow rate of 7.24 g/min and 50 °C. The maximum recovery of SUFA, MUFA, and PUFA was 7.72, 8.65, and 12.70 mg/g, respectively, at extraction (250) bars with a CO_2_ flow rate of 14.48 g/min and 65 °C. Among the tested CO_2_ flow rates, the highest extraction positively influenced the extraction yield of PUFA at all operative pressures and both temperatures, except the test condition at 400 bars and 65 °C. The same phenomena was observed for the recovery of MUFA in comparison to PUFA, while SUFA recovery slightly decreased with an increasing CO_2_ flow rate. However, the lower the CO_2_ flow rate the longer the residence time with the algal cell, which could be helpful in the extraction of SUFA, while MUFA and PUFA extraction may require less contact time with algal cells. Additionally, the lower pressures enhanced the extraction yield of MUFA and PUFA. In contrast, Sánchez-Camargo et al. [54] observed that an increase of the pressure resulted in increased PUFA content, with a maximum of 30% at 370 bars and 57 °C during CO_2_-SF extraction from Brazilian red spotted shrimp waste. Sánchez-Camargo et al. [54] also observed that PUFA concentration may increase by increasing the extraction temperature from 43 °C to 57 °C. Therefore, by combining different sets of pressure and temperature, it is possible to obtain extracts with different compositions [54]. Also, Cheung, [44] reported that, under low pressures, more SUFA are extracted. On the contrary, as the pressure increased, the proportion of unsaturated FAs increased in the extracted phase from *Hypneacharoides* (Red Seaweed). Đurović et al. [32] reported that low pressure and temperature were favorable for the extraction of unsaturated fats and same trend was observed in our study. However, FAs are nonpolar compounds, therefore CO_2_ is a favorable solvent in the extraction of FAs [23,37,38]. 

Results in Figure 3 show that extraction temperature significantly influenced FA recovery at different pressures with a CO_2_ flow rate of 7.24 and 14.48 g/min (Figure 3a–d). Indeed, the extraction of different class of FA, which was reported to be heat sensitive, exhibited a higher extraction at 65 °C and 250 bars with both flow rate in 100 min extraction time. 

### 3.3. Effect of Different Pressure with CO_2_ Flow Rate of 7.24 and 14.48 g/min at 50 °C and 65 °C on EPA Recovery and Purity over Extraction Time

**Figure 4:**
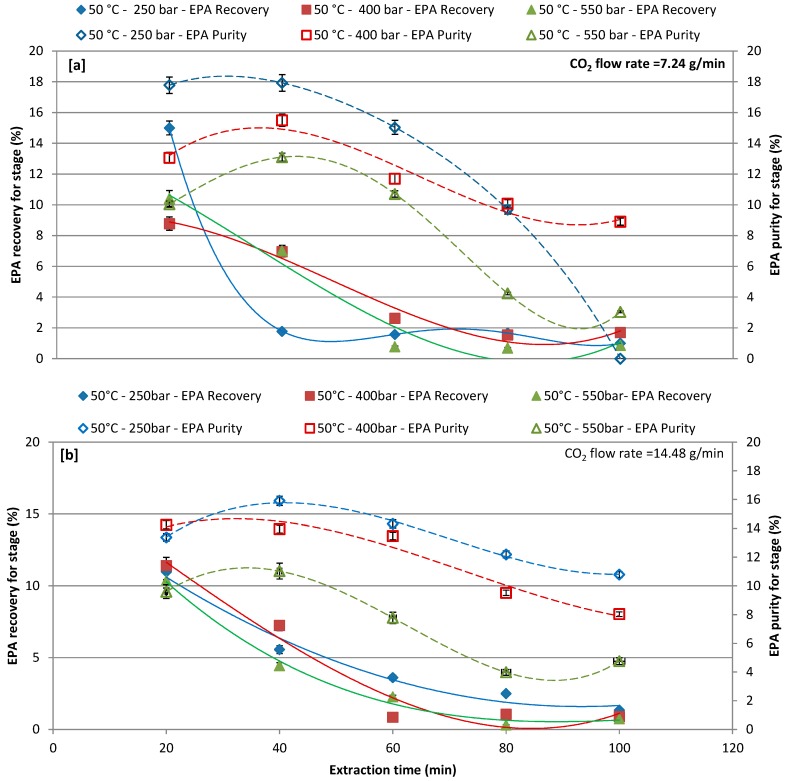
Effect of different pressures on recovery and purity of eicosapentaenoic acid (EPA) at each extraction cycle at 50 °C; (**a**) CO_2_ flow rate of 7.24 g/min; and, (**b**) CO_2_ flow rate of 14.48 g/min.

**Figure 5:**
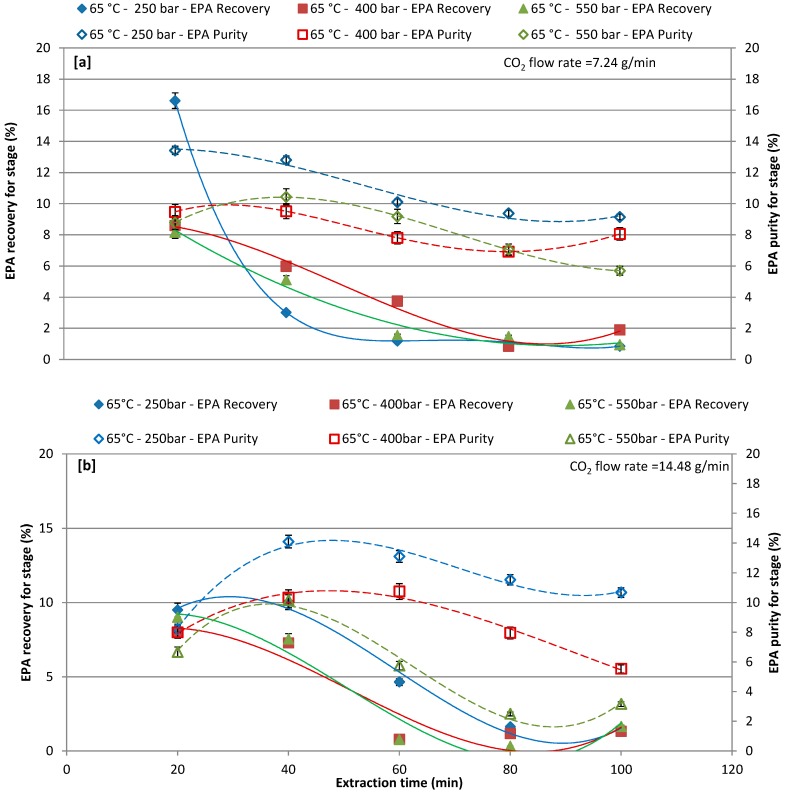
Effect of different pressure on recovery and purity of EPA at each extraction cycle at 65 °C; (**a**) CO_2_ flow rate of 7.24 g/min; and, (**b**) CO_2_ flow rate of 14.48 g/min.

The effects of pressure (250, 400, and 550 bars), as a function of the extraction time, on EPA recovery and purity by CO_2_-SF extraction, were investigated by setting the CO_2_ flow rate at 7.24 and 14.48 g/L and the extraction temperature at 50 °C (Figure 4a,b) and 65 °C (Figure 5a,b). Figure 4a,b report the EPA recoveries and purities at extraction temperature of 50 °C for different extraction pressures and CO_2_ flow rates (Figure 4a,b). In particular, increasing the extraction time the lower CO_2_ flow rate the higher the EPA recovery and purity. For the CO_2_ flow rate of 7.24 g/L results in Figure 4a show that the lowest the pressure (250 bars) the highest the EPA recovery and purity. First extraction cycle (20 min) resulted in a maximum recovery of 15%, 8.77%, and 10.41% at 250, 400, and 550 bars, respectively, while the highest purity was obtained at the second extraction cycle (40 min). The extraction of EPA with a CO_2_ flow rate of 14.48 g/L was less effective at first extraction cycle as compared to a CO_2_ flow rate of 7.24 g/L and same trend was observed for purity. The recovery and purity of extracted EPA gradually decreased by increasing the extraction time. Although reducing the time of extraction might reduce the extraction cost, however, increasing the time of extraction could improve the recovery of target compound, and purity could be achieved [23,55]; therefore, the extraction time is one of the significant factors to be considered when performing CO_2_-SF extraction.

Figure 5a,b report EPA recovery and purity in the extract that was obtained by setting the extraction temperature at 65 °C for different extraction pressures and for different CO_2_ flow rates of 7.24 and 14.48 g/min. For a CO_2_ flow rate of 7.24 g/min, the EPA recovery and purity was affected by the different extractive pressure, the first extraction cycle (20 min) shows around 16.61%, 8.60%, and 8.18% recovery at 250, 400, and 550 bar, respectively, while recovery was gradually decreased with increasing extraction time. By increasing the extraction time, the purity was lightly increases in the first and second extraction cycle, and then started to gradually decrease (Figure 5a). For a CO_2_ flow rate of 14.48 g/min, EPA recovery and purity are generally lower when compared to results that were obtained with CO_2_ flow rate 7.24 g/min. The maximum recovery of 10.03% was obtained at 250 bars at second extraction cycle (40 min). The highest EPA recovery and purity was achieved in first (20min) and second extraction cycles (40 min), respectively, during all operative conditions. Ho et al. [55] stated that the shorter reaction time was beneficial, as it improves the EPA extraction to have lower denaturation effect. 

**Figure 6:**
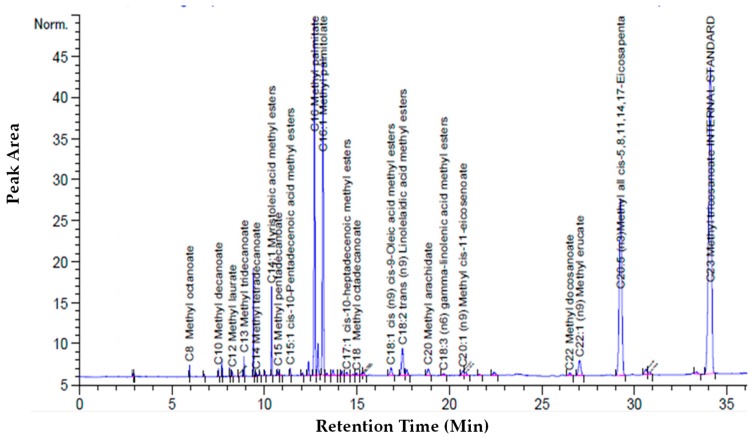
Chromatogram of the first extract at biomass loading of 1.01 g at 250 bars with a CO_2_ flow rate of 7.24 g/min at 65 °C: Chromatogram of first extract.

The cumulative recovery and purity of EPA increased concomitantly with the increase of the CO_2_ flow rate (14.48 g/min). In terms of EPA purity, the highest the CO_2_ flow rate favored a more selective extraction of EPA. The most promising results were obtained by working at 65 °C and 250 bars and the chromatogram of these extracts is shown in Figure 6. 

### 3.4. Effect of Biomass Loading on Cumulative EPA Recovery 

**Figure 7:**
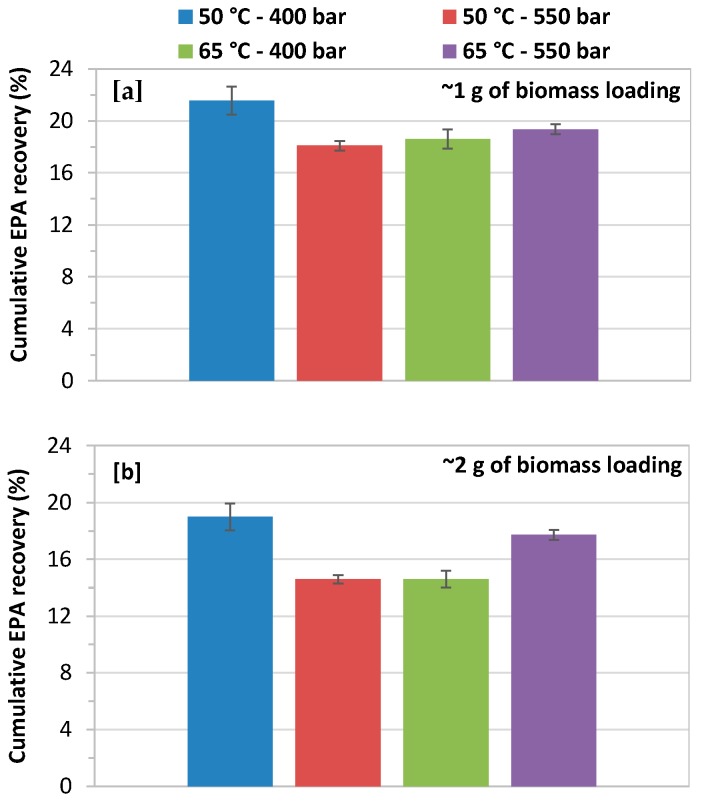
Effect of biomass loading on cumulative EPA yield at 400 and 550 bars with a CO_2_ flow rate of 14.48 g/min at 50 and 65 °C; (**a**) biomass loading of 1.0 g; and, (**b**) biomass loading of 2.0 g.

The biomass loading of 1.0 and 2.0 g had a minor effect on cumulative recovery of EPA, as shown in Figure 7a,b. The high biomass loading significantly decreases the recovery of EPA at 400 and 550 bars with a CO_2_ flow rate of 14.48 g/min at both extraction temperatures of 50 and 65 °C. The highest cumulative EPA recovery, 9.06 mg/g about 22% of theoretical content total EPA (42.28 mg/g), was obtained at 400 bars and 50 °C with biomass loading of 1.0 g. While the lower EPA recovery of 14.6% was achieved at 550 bars and 65 °C with a biomass loading of 2.0 g. 

Awaluddin et al. [56] also reported that long extraction times and high biomass loading negatively affected the carbohydrate recovery. The higher biomass loading had a negative effect on the carbohydrate recovery during subcritical water extraction from *Chlorella vulgaris* [56].

The results of cumulative EPA recovery are collected in Table 3. These results show that a significant variation has been found in the cumulative EPA recovery (6.13–11.50 mg/g). The maximum recovery of EPA 11.50 mg/g was achieved at 250 bars with CO_2_ flow rate of 14.48 g/min and 65 °C. Increasing the CO_2_ flow rate from 7.24 to 14.28 did not show a clear effect on cumulative EPA recovery, while increasing biomass loading negatively influenced the cumulative yield (Table 3). Very similar values were found for the cumulative EPA yield at 400 and 550 bars with CO_2_ flow rate of 14.48 g/min at 65 and 50 °C, respectively with biomass loading of 2 g. These results clearly show that cumulative EPA recovery can be increased by an optimal choice of the extraction conditions. In any case, cumulative EPA recovery was below 25%, therefore more results are required to further increase EPA extraction recovery.

### 3.5. Comparison with Literature for Lipid Recovery Using CO_2_-SF

Literature analysis shows that CO_2_-SF is a suitable technology for lipids extraction. It was reported that CO_2_-SF was selective for neutral lipids, such as triglycerides, but it did not solubilize phospholipids [51,53,57]. Therefore, the lipids that were obtained by CO_2_-SF extraction were mainly composed of triglycerides and a few of other compounds, such as free FAs. Numerous CO_2_-SF, extraction experiments were reported, with extraction conditions and lipid yields being reported in Table 4. The results in Table 4 clearly indicate that varying pressure (200–600 bar), temperature (40–65 °C), CO_2_ flow rate (0.009–166.6 g/min), and extraction time (1–12 h) can modify the extraction capacity of the CO_2_-SF technique. The extraction yield strongly varied with the operating conditions and chemical properties of the used microalgae biomass. It was reported that the lipid extraction yield significantly increased with an increase in pressure at constant temperature; however, the temperature effect was more complex because of the crossover phenomenon, which was well-illustrated in literature [35,58]. Even though the operational conditions were different between one studies and other (Table 4), the results reported in this study are comparable with those reported in literature. 

## 4. Conclusions

The present study focused on lipids extraction from microalgae *N. gaditana* by CO_2_-SF, followed by high-purity EPA recovery. An optimum condition for lipid extraction from microalgae *N. gaditana* with CO_2_-SF was obtained, the maximum lipid extraction is equal to 38.15 mg/g, and it was obtained at 250 bars and 65 °C. The highest EPA purity (~18%) was achieved at first (20 min) and second (40 min) extraction cycle with CO_2_-SF at 250 bars with a CO_2_ flow rate of 7.24 g/min and 65 °C. The greater cumulative EPA yield (11.50 mg/g) was attained at 65 °C and 250 bars, with a CO_2_ flow rate of 7.24 g/min and 1.0 g biomass loading. By changing the extraction conditions, different results have been obtained hence these conditions have to be chosen to maximize the valuable products and to minimize the extraction costs. 

This study provides useful information to optimize EPA production during the growth of algal species. From a commercial perspective, a techno-economic assessment is required and it should ideally be carried out for large-scale extraction where the costs are likely to be very different when compared to the presents laboratory-based study.

## Figures and Tables

**Table 1:** Chemical composition of *N. gaditana*.

Chemical Composition of *N. gaditana*	Concentration (mg/g)
Humidity	47.2
Ash	100.5
Total dietary fiber (TDF)	40.2
carbohydrates	217.4
proteins	470.4
Total lipids	164.5
FAs FAME (mg/g of total lipids)	114.88
	mg/g of FAMEs
Σ SFAs	32.80
Σ MUFAs	29.96
Σ PUFAs	52.12
of which:	
EPA	42.28

Note: Standard deviation was less than 5% in all operative conditions.

**Table 2:** Total extraction and lipid yield from *N. gaditana* at different operative conditions.

Operative Conditions						Total Extraction Yield (mg/g)	Total Lipid Yield (mg/g)
*N. gaditana* Biomass Loading (g)	Bed Height (cm)	*Porosity* [−]	T (°C)	P (Bar)	CO_2_ Flow Rates (g/min)		
1.01	18.6	0.51	50	250	14.48	74.94	31.83
1.01	18.6	0.51	50	400	14.48	68.52	34.46
1.01	18.6	0.51	50	550	14.48	85.21	28.92
1.02	18.6	0.51	50	250	7.24	53.40	31.14
1.01	18.6	0.51	50	400	7.24	72.28	32.24
1.01	18.6	0.51	50	550	7.24	89.60	29.99
1.02	18.6	0.51	65	250	14.48	107.56	38.15
1.01	18.6	0.51	65	400	14.48	92.46	29.64
1.01	18.6	0.51	65	550	14.48	122.96	29.65
1.02	18.6	0.51	65	250	7.24	77.68	34.61
1.01	18.6	0.51	65	400	7.24	101.14	31.79
1.02	18.6	0.51	65	550	7.24	84.78	26.70
2.01	19.6	0.50	50	400	14.48	86.59	28.79
2.01	19.6	0.50	65	400	14.48	66.88	14.93
2.01	19.6	0.50	50	550	14.48	41.38	22.76
2.02	19.6	0.50	65	550	14.48	107.35	26.19

Note: Standard deviation was less than 5% in all operative conditions.

**Table 3:** Effect of pressure (100–550 bar) at 50 °C and 65 °C, extraction time of 110 min) on cumulative EPA recovery (mg/g).

**Operative Temperature (°C)**	**Biomass Loading of 1.01 g**				**Biomass Loading of 2.01 g**			
	**CO_2_ Flow Rate (g/min)**							
	7.24				14.48			
	**Operative Pressure (bar)**							
	**250**	**400**	**550**	**250**	**400**	**550**	**400**	**550**
50	8.46	9.12	8.35	10.14	9.06	7.6	7.97	6.13
65	9.63	8.92	7.3	11.5	7.82	8.13	6.13	7.44

Note: Standard deviation was less than 5% in all operative conditions.

**Table 4:** Comparison of different operative conditions on supercritical CO_2_ extraction of lipids from microalgae.

Species	Biomass Loading (g)	CO_2_ Flow Rate (g/min)	P (Bar)	T (°C)	Extraction Time (min)	Recovery ^#^ (%)	Ref.
*C. vulgaris*	6	30	600	60	180	59.2	[34]
*P. valderianum*	10	~3.6	350	40	90	26.6	[32]
*Nannochloropsis* sp.	1.25	0.62	300	40	160	65.8	[57]
*Synechococccus* sp.	4.6	0.8	400	60	180	6.0	[21]
*C. vulgaris*	150	166.6	280	40	540	31.9	[58]
*Pavlova* sp.	10	na	306	60	360	40.0	[47]
*Tetraselmis* sp.	0.2	0.009	150	40	720	72.3	[19]
*N. gaditana*	1	7.24	250	65	100	23.2	This study

Note: T: Temperature; P: pressure; ^#^ The lipid recovery for CO_2_-SF was calculated based on the initial lipid content of each microalgae; na: not available.
